# Eosinophilic Esophagitis in Pediatrics: The Worst of all Possible Allergy Worlds?

**DOI:** 10.1155/2012/179658

**Published:** 2011-09-14

**Authors:** Russell J. Hopp

**Affiliations:** Department of Pediatrics, Creighton University School of Medicine, Omaha, NE 68131, USA

## Abstract

Eosinophilic esophagitis (EoE) is a relatively uncommon allergic disease. Presenting with variable gastrointestinal symptoms, the definitive diagnosis is made after esophageal visualization and histological confirmation of excessive esophageal eosinophils. The scientific discovery of the pathophysiology of EoE has been aided by its relationship to other common and well-recognized allergic diseases. Similarities and important differences have emerged to distinguish EoE as a significant pediatric allergic disease with unique medical care requirements.

## 1. Introduction

From an early description as a pathological sidebar in 1977 [1] eosinophilic esophagitis (EoE) has become an important allergic disease. Currently, as a pediatric disease entity, EoE requires the diagnostic acumen of multiple specialists, including allergists, gastroenterologists, pathologists, and radiologists. It presents with a spectrum of symptoms depending on age and has a natural history of such short duration that the prognosis for patients and their families is difficult to predict. 

 Eosinophilic esophagitis has the lowest prevalence in the allergic disease family and ranked most to least common: allergic rhinitis, asthma, atopic dermatitis, food allergy, and EoE. Children with EoE often have other preexisting allergic diseases, and, presumptively, young children with EoE likely will develop other allergic disease(s) with age. The pathophysiological understanding of the allergic disease family has grown exponentially in the past decade, which is equally true of EoE [2].

In this paper we will contrast and compare the known pathophysiology and clinical circumstances of each of the allergic diseases and EoE and suggest that EoE has disadvantages to patients greater than or equal to its allergy family members. An excellent consensus paper with recommendations for treating children and adults with EoE has been recently published [3].

## 2. Food Allergy versus Eosinophilic Esophagitis

By every indicator IgE-mediated food allergy has increased in the past decade. In almost all cases, symptoms of IgE-mediated food allergy are readily defined and generally temporally approximate to the food ingested, but not persistent or chronic. The diagnosis is relatively straightforward, using a clinical history supported by appropriate allergy skin tests or specific IgE blood tests. As long as the inciting food is avoided, symptoms are totally gone, and daily therapy is not indicated. With the exception of peanuts and tree nuts, food allergy generally resolves itself by late adolescence. The exact reason for the clinical presence of an allergy to a specific food(s) is still unknown, considering many children allergic to a specific food(s) have concomitant specific food serum IgE levels (or positive skin tests) to tolerated foods.

 In EoE an associated food allergy has been shown to have both pathophysiological and clinical relevance. In contrast to IgE-mediated food allergy where the avoidance of the allergenic food(s) is almost always clinically beneficial, even an elemental diet does not induce remission in all subjects with EoE. And compared to conventional IgE-mediated food allergy, deciding on the food protein avoidance regimen in EoE involves both standard skin testing and in some cases allergy patch testing [4]. The scant data on patch testing suggests that the range of food protein “allergy” in EoE far exceeds conventional IgE-mediated food allergy mechanisms. Despite the information gained using these techniques (skin testing and patch testing) in some situations complete food protein avoidance is necessary for effective control of symptoms [5]. And finally, in contrast to food allergy, current evidence does not support the eventual tolerance of allergic food(s) and the total resolution of EoE [6]. Although morbidity with EoE is relatively high, mortality, as compared to food allergy, has not been reported.

## 3. Eosinophilic Esophagitis and Atopic Dermatitis

Atopic dermatitis (AD) pathophysiology is a complex interaction of intrinsic dermal/epidermal dysfunction and humoral (IgE) and cellular (T-cell) reactivity to the environment. In particular, abnormal filaggrin protein(s) (an epidermal component) has been linked to severe AD phenotypes [7], with a resultant loss of epidermal integrity, thus allowing interaction between the external environment and the heightened immune response. Recent research has suggested a diminished filaggrin protein in EoE, resulting in decreased esophageal barrier function [8].

 Atopy is a common finding in pediatric atopic dermatitis and EoE, although nonatopic forms of AD and EoE are not uncommon. IgE-mediated sensitization to food protein(s) is commonly enhanced in both disease conditions, and the elimination of sensitized foods has been a cornerstone of therapy in EoE and a frequent discussion point in AD. Multiple studies investigating food avoidance as an effective therapy for AD have not been convincing, despite the fact that IgE-mediated food sensitization is common in AD.

 The clinical relevance of avoiding IgE-mediated sensitized foods in AD patients is limited. Preceding the use of patch testing in EoE for foods was a movement to isolate clinically relevant food allergies in AD using a delayed (48–72 hours) food response [9]. Although in limited use currently, these strategies suggest a more complex immune response to food protein than through a Type 1 immune reaction in both EoE and AD. The limited studies of patch testing in EoE suggest a stronger association with disease activity and/or therapeutic responsiveness to certain food protein, especially milk [10]. Also, food protein exposure in AD has several pathways to sensitization, including contact, airborne, and even transplacental or via breast milk, while in EoE food protein exposure may be through a systemic route with esophageal transmural migration, or possibly by direct contact during food ingestion. 

 Environmental allergen sensitization is common to both AD and EoE, but studies do not exist that compare rates of environmental sensitization in children with a single disease state (EoE or AD), or each disease at different ages. It is generally held that young AD patients eventually develop environmental allergen sensitization and often second or third allergic diagnoses, but similar studies showing the development of other allergic diseases have not been done with early onset EoE. Conventional allergy immunotherapy, often issued in allergic rhinitis and allergic asthma, is not commonly utilized for AD and has not been reported for EoE.

 The prognosis of AD in children is considered good, with many children having resolution of active disease by adolescence. The current longitudinal data for pediatric-onset EoE from a single center was not optimistic for improvement [6], strongly suggesting a persistent course, possibly into adulthood.

## 4. Eosinophilic Esophagitis versus Allergic Rhinitis

 Allergic rhinitis is a relatively straightforward and common allergic disease which usually starts in childhood. The exposure and sensitization to an airborne allergen is then recentered to an intranasal allergy response on re-exposure. Although not commonly emphasized, the late phase allergic is largely responsible for chronicity of symptoms. Subepithelial thickening and remodeling are apparently present, but do not usually receive the same consideration as do the same processes in the lower airway.

 Food allergens only extremely rarely are involved in allergic rhinitis, although a corollary process, pollen-food allergy syndrome, may occasionally coexist. Allergic rhinitis therapy is directed by and enhanced with appropriate skin testing, with good clinical correlation. Avoidance can be helpful, topical therapy with intranasal corticosteroids is often successful, and allergy immunotherapy is a mainstay. Complications arising from allergic rhinitis are uncommon, but life-long disease is common.

 In comparison to allergic rhinitis, EoE is uncommon, sometimes indolent, or under recognized, with only an occasional association with seasonal worsening. Eosinophilic esophagitis patients can have positive skin tests to both seasonal and nonseasonal allergens, but clinical esophageal symptomatology to environmental allergens, in comparison to nasal symptoms as seen with allergic rhinitis, is less obvious. Food allergy, both immediate and possibly non-IgE mediated, is common to EoE, unlike allergic rhinitis.

 Recent data strongly suggests a significant role for remodeling/fibrosis in EoE [11] and even smooth muscle hyperplasia and possibly hyperresponsiveness [12]. Therapy for EoE often hinges on topical inhaled corticosteroids, but avoidance of food allergens can be partially (or even near totally) remissive. Immunotherapy for EoE has not been studied to date, and, in large part, therapeutic regimens are empiric, without the long time honored benefit of placebo-controlled trials required in new allergic rhinitis therapies. Like allergic rhinitis, the pediatric EoE patient may have long-lasting disease, although the opportunity for serious, life-altering sequela is likely much higher in EoE.

## 5. Eosinophilic Esophagitis versus Asthma

The occasionally used term “asthma of the esophagus” places EoE in the same realm of disease pathophysiology of the frequently cited “most common chronic disease of children” asthma if not so much for its frequency but to its impact on morbidity. The rapidly advancing basic research studies in EoE, with a several decades of asthma pathophysiology as a guide, have quickly moved EoE into position of significant biological complexity.

 Asthma has run a gamut of pathophysiological causes, smooth muscle constriction, bronchial hyperresponsiveness, eosinophilic inflammation, specific T-cell hyperactivity, and extensive cytokine production, although many of the processes currently recognized date to work by Slater in the early 1800s and Osler by the turn of the century [13]. EoE was first described only 4 decades ago. Like asthma, EoE has a hierarchy of recognized histological consequences, the discovery of which has been compressed into a mere decade of research.

 Asthma in young children is usually associated with nonallergic triggers, especially viral illnesses, while asthma that continues into grade school and later-onset asthma usually has allergic comorbidity. Food allergy is a frequent comorbid process, but avoidance of food protein never induces remission of active disease. Asthma is usually associated with wheezing or recurrent cough and is usually considered early after its onset. The presence of wheezing is readily apparent to most parents and is a tell-tale symptom for health care providers. The rapid response to beta-agonists in a clinical setting, or in a pulmonary function laboratory, provides confirmation of likelihood for asthma. Current asthma guidelines provide a literature-supported, evidence-based approach for medical and supportive management, with advancing complexity for difficult-to-manage patients. Unfortunately, asthma has always maintained an imposing morbidity among the allergic diseases, although the rate of asthma deaths has decreased this decade. The natural history of pediatric asthma supports a reasonable chance for sustained remission, although fixed airway flow changes may persist. 

 EoE has a spectrum of presentations in children, with a different face with advancing age of onset. No one symptom clearly dominates, unlike the recurrence of wheezing or chronic cough seen in asthma. The relative infrequency, as compared to asthma, may delay the diagnosis. In some situations, the presence of EoE is totally unknown until an incidental finding on an endoscopy for an unrelated concern or only after the retrieval of a stuck esophageal foreign body. The incidence of “silent” EoE is unknown. In contrast the medical literature has thousands of epidemiological studies, using multiple methods of ascertainment, looking at “hidden” asthma. Unlike asthma, the definitive diagnosis requires an invasive procedure, with the subsequent initiation of largely empirical medical and/or food protein avoidance therapy(s). Any prescribed therapeutic intervention does not generally allow for a certain response, or easily determined benefit, without a repeat endoscopy. A published report in pediatric EoE patients followed for over one decade does not support a reasonable chance of remission [6]. Mortality from eosinophilic esophagitis has not been reported, in contrast to asthma, currently at over 3000 deaths per year.

## 6. Summary

Eosinophilic esophagitis certainly matches the biological complexity of atopic dermatitis and asthma. Asthma, allergic rhinitis, food allergy, and to a less extent atopic dermatitis, have long recognized and/or easily undertaken medical protocols, which are further supported by evidence-based research. The long-term clinical impact of EoE is yet, largely, unknown, but studies suggest extended chronicity, while the other allergic diseases, especially atopic dermatitis and most food allergies, have a very reasonable likelihood of remission. Subject to some debate, the incidence of EoE is likely increasing, as are all other allergic diseases. The potential for morbidity in EoE appears to be high, but without current reports of mortality, unlike food allergy or asthma. Overall, EoE is a complex allergic disease, with long-term concerns, *with minimal pediatric placebo-controlled trials* [14, 15] to guide therapy, and no readily apparent method of following clinical progress, except repeated invasive testing procedures. Extensive information has been gained on its pathophysiology, *aided* by years of sequential understanding of the other allergic diseases.
